# Real Time Monitoring of Calcium Oxalate Precipitation Reaction by Using Corrosion Resistant Magnetoelastic Resonance Sensors

**DOI:** 10.3390/s20102802

**Published:** 2020-05-14

**Authors:** Beatriz Sisniega, Ariane Sagasti Sedano, Jon Gutiérrez, Alfredo García-Arribas

**Affiliations:** 1 Departament de Electricidad y Electrónica, Universidad del País Vasco/Euskal Herriko Unibertsitatea (UPV/EHU), Barrio Sarriena s/n, 48940 Leioa, Spain; ariane.sagastis@ehu.eus (A.S.S.); jon.gutierrez@ehu.eus (J.G.); alfredo.garcia@ehu.es (A.G.-A.); 2 BC Materials, Basque Center for Materials, Applications and Nanostructures, UPV/EHU Science Park, 48940 Leioa, Spain

**Keywords:** magnetoelasticity, precipitation, mass measurement, chemical sensor

## Abstract

The magnetoelastic resonance is used to monitor the precipitation reaction of calcium oxalate ($CaC_{2}O_{4}$) crystals in real-time, by measuring the shift of the resonance frequency caused by the mass increase on the resonator. With respect to previous work on the same matter, the novelty lies in the adoption of an amorphous ferromagnetic alloy, of composition $Fe_{73}Cr_{5}Si_{10}B_{12}$, as resonator, that replaces the commercial Metglas^®^ 2826 alloy (composition $Fe_{40}Ni_{38}Mo_{4}B_{18}$). The enhanced corrosion resistance of this material allows it to be used in biological environments without any pre-treatment of its surface. Additionally, the measurement method, which has been specifically adapted to this application, allows quick registration of the whole resonance curve as a function of the excitation frequency, and thus enhances the resolution and decreases the detection noise. The frequency shift is calibrated by the static deposition of well-known masses of $CaC_{2}O_{4}$. The resonator dimensions have been selected to improve sensitivity. A 20 mm long, 2 mm wide and 25 μm thick magnetoelastic resonator has been used to monitor the precipitation reaction of calcium oxalate in a 500 s time interval. The results of the detected precipitated mass when oxalic acid and calcium chloride are mixed in different concentrations (30 mM, 50 mM and 100 mM) are presented as a function of time. The results show that the sensor is capable of monitoring the precipitation reaction. The mass sensitivity obtained, and the corrosion resistance of the material, suggest that this material can perform excellently in monitoring this type of reaction.

## 1. Introduction

Magnetoelastic resonance sensors are typically made of amorphous ferromagnetic ribbons, with good values of spontaneous magnetization and saturation magnetostriction, and low magnetocrystalline anisotropy [1,2,3]. In this type of material the mechanical and magnetic properties are intimately coupled by magnetostriction, and so an acoustic wave can be excited within the material by the application of an alternating magnetic field. Reciprocally, mechanical disturbances, such as oscillations, can be magnetically detected by the voltage induced in a coil in the proximity of the sample. The magnetoelastic material, which is usually fabricated in the form of a thin ribbon, can enter in resonance at certain frequencies of excitation, compatible with the dimensions and elastic properties of the material. The resonance is extremely sensitive to different external parameters, which can be used to design different types of sensors [4,5]. In particular, differences in mass loading (∆m) cause a variation of the resonance frequency ($∆f=f_{r}-f_{0})$ of a bare magnetoelastic ribbon of mass $m_{0}$, determined by the expression [5]:

(1) $$\Delta f=-\frac{1}{2}f_{0}\left(\frac{\Delta m}{m_{0}}\right)$$

The sensitivity to these external parameters, together with the ability to query and detect remotely, make these devices especially interesting for sensing biological and chemical agents.

Previous works by Boropoulos and co-workers [6] have used magnetoelastic sensors, based on the commercial material Metglas^®^ 2826, for monitoring the kinetics of different precipitation reactions, such as the precipitation of calcium oxalate ($CaC_{2}O_{4}$) crystals, one of the most common minerals that form calcifications in the urinary tract (so-called kidney or bladder stones). In humans, there are essential inorganic salts for diverse metabolic activities, like sodium chloride (NaCl), calcium chloride ($CaCl_{2}$), magnesium chloride ($MgCl_{2}$), sodium bicarbonate ($NaHCO_{3}$), potassium chloride (KCl), sodium sulfate ($Na_{2}SO_{4}$), calcium carbonate ($CaCO_{3}$), and calcium phosphate ($Ca_{3}{\left(PO_{4}\right)}_{2}$). These inorganic salts dissociate in solution into ions (or electrolytes). If some of these ions are not properly absorbed within the body, they will tend to crystallize in small grains or stones.

Concerning kidney stones, their formation is the result of a complex physicochemical process that leads to crystallization. The stones, also known as renal calculi, are mostly calcium based [7]: calcium oxalate accounts for approximately 60% to 70%, struvite (magnesium ammonium phosphate) for 10% to 20%, uric acid for 5% to 10%, and in low quantities also cystine (<1%) and calcium phosphate (<5%). Some conditions, such as hypercalciuria or hyperoxaluria, can contribute to increasing the risk of suffering this pathology. Hypercalciuria is the result of an increase in the quantity of filtered calcium and a decrease of its re-absorption in the kidney. It is thought to contribute to kidney stone-formation by creating a urine supersaturated with respect to calcium. Supersaturation arises from a concentration above a material’s solubility in water, and leads to the formation of crystals. In a urine sample collected over 24 h from an average adult, a quantity of 100–250 mg of calcium is expected. In conditions of hypercalciuria the urine calcium excretion is greater than 275–300 mg/day in men, or 250 mg/day in women. Hypercalciuria can also be defined as a daily urinary excretion of more than 4 mg calcium/kg body weight [8,9]. In the adult population, 5% of men have hypercalciuria and, of them, about 10% will form a kidney stone. Urine has a remarkable ability to inhibit calcium crystallization, which thus prevents most of the population from continuously forming such stones. Hyperoxaluria, on the other hand, is a metabolic disorder of increased excretion of oxalate in urine, and it is also related to the formation of stones in the urinary tract [10]. The normal values of oxalate excretion in urine are under 40 mg/day [11]. Urine calcium oxalate concentration values up to 100 μmol/L are reported in patients suffering from primary hyperoxaluria [12].

The use of magnetoelastic sensors to remotely monitor these types of reactions (deposition of crystallites) can provide fundamental information about the precipitation process in biological fluids. It can increase our understanding of these complex processes of bio-mineralization since this technique allows, for example, to study precipitation systems under the influence of different factors (such as pH, or concentration and chemical composition of the urine), to enhance our knowledge about which factors or substances favour or inhibit the formation of crystals in the urinary tract [13,14]. Information about the precipitate mass in real-time can complement the information (usually obtained by monitoring changes in pH or concentration) of studies in artificial systems that mimic the human physiological conditions, like human urine [14].

In the mentioned work [6], the formation of the insoluble salt crystals was tracked by the changes in the resonant frequency of the magnetoelastic sensor, which decreases as the precipitate is deposited on its surface, as a direct consequence of the increase of the total mass of the resonant strip. In spite of the excellent results published in that work, there are two main aspects that, in our opinion, can be improved. First, the Metglas^®^ 2826 alloy used as magnetoelastic resonator had to be submitted to a previous conditioning process to protect it from corrosion occurring in the biological medium. Second, the resonance changes due to precipitation were followed by measuring the voltage induced at a fixed frequency (117 kHz, above the resonant frequency), in order to be able to follow the process in real-time, a procedure that reduces the accuracy and resolution of the measurement.

In the present work, we have reproduced the process of monitoring the precipitation reaction of calcium oxalate crystals, but in this case, we have used an amorphous ferromagnetic alloy of composition $Fe_{73}Cr_{5}Si_{10}B_{12}$ as resonator. This alloy does not need any special pre-treatment, since it has been already demonstrated to exhibit excellent resistance to corrosion [15,16]. Additionally, the measurement process has been optimized, so we can measure the complete resonance curve fast enough to directly track the evolution of the resonance frequency along the precipitation process. The change in the resonance frequency during the precipitation process has been directly correlated to the amount of precipitated material on the resonator through a calibration with known deposited masses. We have analyzed carefully the validity of this calibration, and concluded that, even though the calibration is performed with the resonator vibrating in air, it provides a valid estimation of the mass of precipitated materials in the liquid phase.

## 2. Materials and Methods

### 2.1. Magnetic and Magnetoelastic Materials Characterization

The magnetoelastic ribbon of composition $Fe_{73}Cr_{5}Si_{10}B_{12}$ used in this work was kindly provided by Vacuumschmelze GmbH & Co., KG, Germany. This composition contains a small amount of chromium (5% atomic) that allows the formation of a passivation layer on the material, thus favoring its corrosion-resistant behavior. We selected the geometry of the strips, cut from a larger ribbon, to be 20 mm × 2 mm × 25 µm, with a length to width ratio (*R* = *L*/*w* = 10), high enough to ensure a good magnetoelastic coupling [17] and enough surface for the deposition of precipitate.

Magnetic and corrosion resistance characterization appear extensively explained in [16]. Magnetoelastic measurements, for the real-time monitoring of the resonance frequency, were carried out by using a homemade experimental set-up [18]. Briefly, the system used to register the resonance–antiresonance curve consists of three coaxial solenoids: one to apply the constant bias field (H); a second one to produce the alternating field to magnetostrictively excite the sample; and the third one, consisting of a compensated pick-up coil, to detect the induced magnetization oscillations, from which the frequencies of the corresponding magnetoelastic resonance (*f_r_*) and anti-resonance (*f_a_*) are determined. We optimized the data acquisition procedure to be able to register the whole resonance–antiresonance curve quick enough to follow the precipitation process. A spectrum analyzer (HP 3589A) working in swept mode was used to produce the excitation and to receive the signal induced in the pick-up coil. The range of the frequency sweep was kept as small as possible. It was determined prior to the experiment, based on the expected change of the resonance frequency. The speed of the sweep was set as fast as possible, while maintaining a compromise with the quality of the registered curve. The goal was to allow discriminating differences in the resonance frequency of 100 Hz. After recording the resonance–antiresonance, the frequency of the maximum and its amplitude were measured using the built-in analysis procedures of the analyzer, and transmitted to a control computer. The typical time to record a whole magnetoelastic resonance curve was about 5 s.

The measured resonant frequency (*f_r_*) varies with the bias field *H*, since it is directly related to Young’s modulus as $f_{r}=\left(\sqrt{E\left(H\right)/\rho }\right)/2L$ [19], where *L* and *ρ* are the length and density of the ribbon shaped material. The field-dependence of the elastic modulus is known as *ΔE* effect and quantified as $\Delta E\left(\%\right)=(1-E\left(H\right)/E_{S})\times 100$, *E_S_* being the Young’s modulus measured at magnetic saturation. Other important information that can be determined from these measurements are the magnetoelastic coupling coefficient ($k^{2}=\left(\pi^{2}/8\right)\left(1-{\left(f_{r}/f_{a}\right)}^{2}\right)$) [20] and the quality factor of the resonance $\left(Q=f_{r}/∆f\right)$, all quantities being functions of the applied external magnetic field.

Table 1 shows the measured magnetic, magnetoelastic and corrosion resistance properties of the $Fe_{73}Cr_{5}Si_{10}B_{12}$ ribbon used in our calcium oxalate detection experiments. 

### 2.2. Sensor Calibration

**Figure 1:**
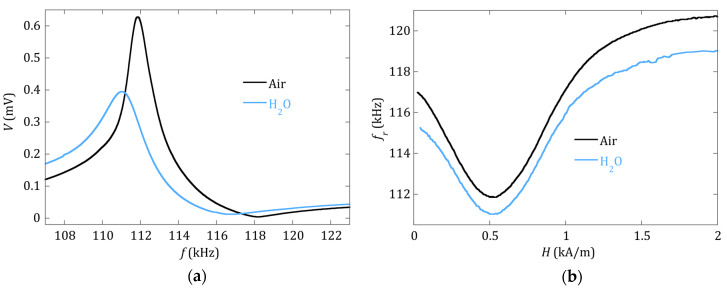
(**a**) Magnetoelastic resonance curves measured at *H* = 517 A/m, and (**b**) dependence of the resonant frequency ($f_{r}$) with the applied magnetic field, for the $Fe_{73}Cr_{5}Si_{10}B_{12}$ strip measured in air and when it is immersed in distilled water.

Prior to the calibration of the sensor, we studied the influence that the medium, in which the magnetoelastic strip is immersed (air or distilled water), has on the observed magnetoelastic resonance. Figure 1 shows both the change in one single magnetoelastic resonance curve, and over the whole measured *f_r_*(*H*) dependence, from zero to saturation applied *H* magnetic fields.

As expected, and due to the differences in viscosity and density of air and distilled water, the effect of the immersion in water is to increase the damping in a uniform way over the whole strip. This means that, at the same applied bias field *H*, the magnetoelastic resonance curve widens (the quality factor *Q* value decreases) and the resonance frequency decreases (see Figure 1a). The *f_r_*(*H*) curves displayed in Figure 1b show that the decrease of the resonance frequency is almost the same at any applied *H* magnetic field, at least in the vicinity of the minimum, which is where the measurements are made during the precipitation experiment.

The calibration of the sensor sensitivity to added mass was performed by depositing in successive steps a known precipitated mass on the sensor and measuring the corresponding change in its magnetoelastic resonance frequency. The deposition process for the calibration is the same used for the calcium oxalate detection measurement conditions: the sensor is immersed in a small vial with a mixture of the precipitation solution with different concentrations, and once the precipitated crystallites are deposited onto it, it is taken out of the vial carefully and dried. Afterwards, the sensor is weighed on a precision balance (0.1 µg resolution), and its magnetoelastic resonant frequency is measured at a bias field corresponding to the minimum of the *f_r_*(*H*) curve. This minimum corresponds to a bias field equal to the anisotropy field (*H_k_*) of the resonator, which in our case is *H_k_* = 517 A/m. At this anisotropy field value, the sensitivity of the magnetoelastic resonance frequency to the amount of added mass is maximum [23].

For this calibration, Equation (1) is just an approximation of the more general expression [5]:

(2) frf0=(1+∆mm0)−12

The mass and resonance frequency of the bare magnetoelastic sensor strip are *m*_0_ = 7.6063 mg and *f*_0_ = 115.38 kHz, respectively. The mass changes suffered by the sensor during the calcium oxalate precipitation process are greater than 5% of that initial bare weight. Therefore, a second order expansion of Equation (2) has been used to obtain an appropriate fit for the calibration curve [24]:

(3) $$∆f=f_{r}-f_{0}=-\frac{f_{0}}{2m_{0}}∆m+\frac{3f_{0}}{8m_{0}^{2}}{\left(∆m\right)}^{2}=a_{1}\Delta m+a_{2}{\left(\Delta m\right)}^{2}$$

Following this procedure, the mass calibration allows a quantitative knowledge of the mass of precipitate deposited on the sensor during the precipitation process, and therefore, a real-time monitoring of the reaction kinetics.

**Figure 2:**
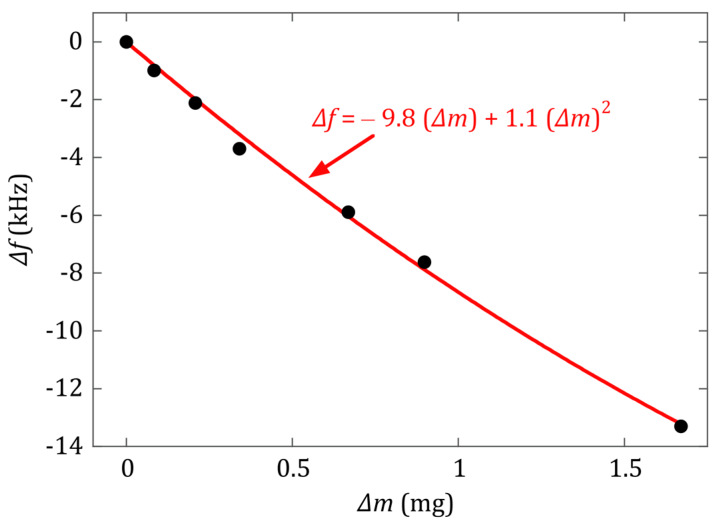
Calibration curve obtained from the changes in the resonance frequency of the $Fe_{73}Cr_{5}Si_{10}B_{12}$ resonator (measured in air), caused by different calcium oxalate mass depositions on its surface. Black dots represent the measured calibration points. The solid red line represents a fit to the second order expression of Equation (3), with coefficients *a_1_* = −9.8 kHz/mg and *a_2_* = 1.1 kHz/mg^2^.

The obtained calibration curve can be seen in Figure 2, and the experimentally obtained calibration constants are $a_{1}=-9.8\;\pm 0.4\;\mathrm{kHz}/\mathrm{mg}$ and $a_{2}=1.1\;\pm 0.3\;{\mathrm{kHz}/\mathrm{mg}}^{2}$. It is to be noted that, for the calibration, the measurement of the frequency shift caused by the precipitated mass on the resonator is made in air, whereas in the real-time experiments described in Section 2.3, the resonance takes place inside the water-filled vial. As evidenced in Figure 1b, the resonance frequency in water is systematically lower than the one in air. Grimes et al. [4] gave already an expression for the observed decrease in the magnetoelastic resonance frequency when the vibrating sensor is immersed in a viscous liquid:

(4) ∆f=−πηρl2πdρs(f0)12

In our case, $\eta =0.89\times 10^{-3}\;\mathrm{Pa}\cdot s$ and $\rho_{l}=1000{\;\mathrm{kg}/m}^{3}$ are the viscosity and density of water, and $\rho_{s}=7200{\;\mathrm{kg}/m}^{3}$ and d=25 μm are the density and thickness of the resonant strip, respectively. If we apply the frequency decrease established by Equation (4) to the resonance frequency values in the calibration curve, we obtain the same values, within the error, of the fitting parameters $a_{1}$ and $a_{2}$. Therefore, we conclude that the calibration curve measured in air is a good approximation to determine the amount of calcium oxalate mass deposited in the real-time experiments, when the resonant strip is immersed inside the solutions.

**Figure 3:**
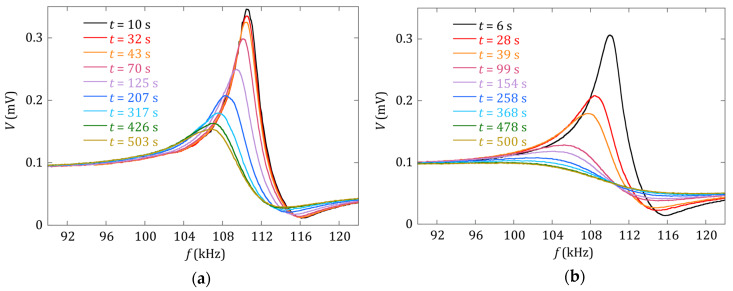
Measured magnetoelastic resonance curves of the sensor at different times during the precipitation process for solutions of oxalic acid and calcium chloride with concentrations of (**a**) 30 mM and (**b**) 100 mM.

Finally, the theoretical values of the mass calibration constants appearing in Equation (3) can be calculated to be $a_{1}=-\frac{f_{0}}{2m_{0}}=-7.6\;\mathrm{kHz}/\mathrm{mg}$ and $a_{2}=\frac{3f_{0}}{8m_{0}^{2}}=0.8{\;\mathrm{kHz}/\mathrm{mg}}^{2}$. It can be observed that the experimental calibration constants are both 22%–27% higher than these expected values. This kind of discrepancy is observed in other works [24,25]. The origin of the deviation is not clear, but can be related to two aspects: first, an original oversimplification in the derivation of Equation (2), that identifies the increase of mass as a change in the resonator density [5]. Second, the fact that the apparent resonance frequency (maximum of the resonance curve) is shifted to lower frequencies when the damping in the resonance is increased. Equation (2) does not consider this effect; however, experimental evidence shows that mass loading increases the damping of the resonance (as clearly shown in Figure 3). Comparing with the sensor sensitivity reported by Bouropoulos et al. [6], −1.38 kHz/mg, the main mass calibration constant ($a_{1}$) obtained for the $Fe_{73}Cr_{5}Si_{10}B_{12}$ magnetoelastic resonator in this work is about seven times more sensitive. The main reason that accounts for this fact is the better magnetoelastic coupling coefficient (k) of the $Fe_{73}Cr_{5}Si_{10}B_{12}$ alloy than the Metglas 2826 one, as can be seen in the characteristics given in Table 1. This is directly related to the length-to-width ratio (R=L/w) chosen for the resonator used in our experiments (R=10, instead of R~3 of the previous work [6]). According to [17], the magnetoelastic coupling coefficient reaches its optimal value at R≥12, so in our case the aspect ratio is high enough to ensure good magnetoelastic properties and quality factor (Q) of the sensor.

### 2.3. Calcium Oxalate Precipitation

The monitoring of the precipitation reaction was carried out by placing the magnetoelastic strip inside a vial and mixing equal parts of solutions (0.6 mL each) with the same concentration of oxalic acid and calcium chloride, leading to the formation of the insoluble crystals:

$$CaCl_{2}\left(aq\right)+H_{2}C_{2}O_{4}\left(aq\right)\overset{\;}{\to }\;CaC_{2}O_{4}\left(s\right)+2HCl\left(aq\right)$$

Solutions of different concentration (30 mM, 50 mM and 100 mM) were used in order to observe its effect on the rate of reaction, and to assess the detection capability of the technique. Precipitation of the calcium oxalate salt will occur immediately after the mixing of the solutions if, with respect to the solid phases, the resulting solution concentration turns out to be supersaturated (SI > 0). SI denotes the saturation index of a sparingly soluble salt in an aqueous medium [26,27]. On the contrary, precipitation will not occur for undersaturated (SI < 0) concentrations. 

Prior to the time monitoring of the calcium oxalate salt precipitation process, a control curve of the sensing strip within the vial with only distilled water was also performed.

## 3. Results and Discussion

**Figure 4:**
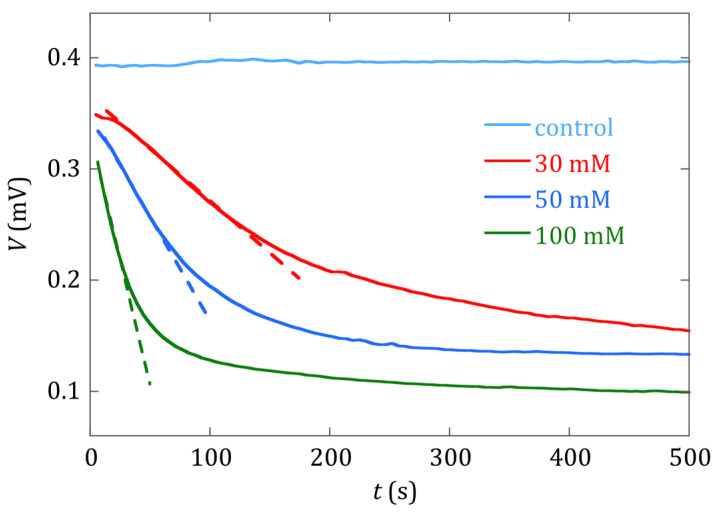
Temporal evolution of the amplitude (mV) of the measured signal (resonance amplitude), as the precipitation reaction progressed and precipitate crystals were deposited on the sensor surface. Curves are shown for different reactant concentrations (30 mM, 50 mM and 100 mM), and a control test (sensor in a vial with distilled water). Linear fits of the initial slope of each voltage curve are shown in dashed lines.

The effect of the different concentrations of the prepared precipitation solutions can be directly inferred from Figure 3 and Figure 4. Figure 3 shows, in the same time window from 10 to 500 s, how quickly both the magnetoelastic resonance frequency (in kHz) and the amplitude of the detected signal (in mV) decreased as the concentration of the constituents within the solutions increased. The rate at which this decrease occurred was clearly higher for the 100 mM than for the 30 mM concentration solution.

Figure 4 shows the temporal evolution of the amplitude of the detected signal, as the precipitation reaction occurs. The linear fit of the initial slope of each measured curve indicates how fast the deposition process took place. In our study, and for the 30, 50 and 100 mM concentration cases, we got a signal decrease ratio of −0.95 μV/s, −1.76 μV/s and −4.5 μV/s, respectively. These ratios reflect how the precipitation process of the calcium oxalate took place, indicating that it was clearly different between the 30 mM concentration solutions and the 50 and 100 mM concentration ones. For these last two cases, there was a fast decrease in the measured signal, motivated by the high supersaturated character of both solutions, and subsequent spontaneous and quick formation of the salt. The higher the slope of the initial part of the curve, the faster the precipitation process took place.

As remarked previously by other authors, when the precipitation of the salt happens, crystal growth is prevalent with receding nucleation [28]. In a final step, the decrease of the supersaturation character of the solutions leads to a plateau-like regime, with ratios −17.1 nV/s and −29 nV/s for the 50 and 100 mM concentration cases (300 s < time window < 500 s), indicating that the calcium oxalate precipitation process is practically finished.

On the contrary, for the 30 mM concentration case, the measured amplitude of the detected signal of the sensor does not show the previously described behavior. This curve shows a monotonous and smooth decrease with time, with the lowest initial slope or precipitation rate, and no plateau regime is observed for the same time window (500 s) used for the other two concentration cases. In fact, the signal decrease ratio is now −0.13 μV/s (300 s < time window < 500 s), just slightly lower than in the initial step of the deposition process.

**Figure 5:**
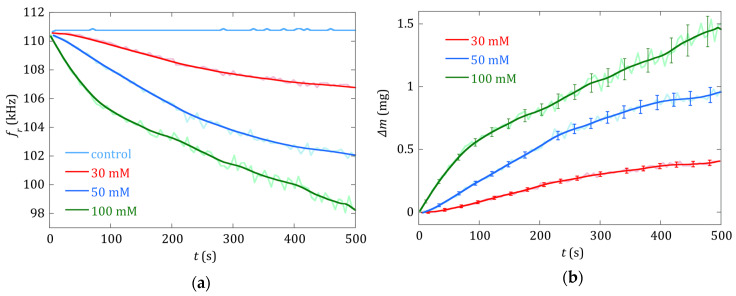
(**a**) Change of the magnetoelastic resonance frequency measured during the precipitation process for different reactant concentrations (30, 50 and 100 mM) and for the control test curve (sensor in a vial with distilled water). (**b**) Deposited mass of calcium oxalate crystals on the sensor during the reaction of precipitation for different reactant concentrations (30 mM, 50 mM and 100 mM), with the mass determination error. The actual measurements are plotted in light color. Intense solid curves correspond to smoothed data.

Finally, the change in the detected magnetoelastic resonance frequency, and the subsequently determined mass load, for the three solution concentrations presented in this study are shown in Figure 5. The deposited mass values were calculated from the resonance frequencies by applying the calibration expression given in Equation (3), using the fitting coefficients from Figure 2. The error bars in the figure are calculated as:

(5) $$\varepsilon_{∆m}=\sqrt{{\left(\frac{\partial ∆m}{\partial a_{1}}\varepsilon_{a_{1}}\right)}^{2}+{\left(\frac{\partial ∆m}{\partial a_{2}}\varepsilon_{a_{2}}\right)}^{2}+{\left(\frac{\partial ∆m}{\partial ∆f}\varepsilon_{∆f}\right)}^{2}}$$

where $\varepsilon_{a_{1}}$ and $\varepsilon_{a_{2}}$ are the errors obtained by the fitting procedure in Figure 2, and $\varepsilon_{∆f}$ is the experimental uncertainty in the resonance frequency ($\varepsilon_{∆f}\;$= 100 Hz).

Figure 5 shows curves that evolve in a monotonous way in all cases, showing that the increase of the deposited mass of calcium oxalate onto the magnetoelastic strip leads to a decrease in its measured resonance frequency. However, the evolution of the curves appearing in Figure 4 and Figure 5 is different, and hints at the complementarity of the information given by each one: while the decrease of the amplitude of the magnetoelastic resonance in the detected signal gives information about how fast this process occurs and eventually finishes, the decrease in the measured magnetoelastic resonance frequency value gives information about the amount of mass deposited along this process, by using the corresponding calibration equation.

Related to the practical application of our detection system, a targeted calcium oxalate concentration of 100 μmol/L = 12.8 mg/L (considering this value as a risk value for forming stones) translates to 0.015 mg = 15 μg of $CaC_{2}O_{4}$ to be detected in our 1.2 mL solution vial. According to the sensor calibration previously obtained (Section 2.2), a mass load of 0.015 mg of calcium oxalate on the resonator produces a magnetoelastic resonance frequency change of 0.15 kHz, just in the theoretical limit of the detectable change for our experimental set-up. Considering the minimum change in resonance frequency that we can discern (100 Hz), and the calibration constants obtained, the minimum theoretical deposited mass that our system is capable of detecting is 0.01 mg = 10 μg. However, the noise of the measured resonant frequency curves, up to 1 kHz (Figure 5a), raises this detection limit to 0.1 mg = 100 μg.

The results presented in this study demonstrate the feasibility of using magnetoelastic sensors in those cases where remote and non-destructive detection is required. This is the case for most biological or chemical agent detections. It turns out to be also a fast detection technique, that can provide accurate results in short time windows, of the order of several minutes. These rapid measurements are useful for monitoring this type of precipitation reaction, which can be quite fast, with sufficient accuracy.

Future work has to address the increase of the sensitivity of our magnetoelastic sensor, by reducing both the resonance frequency discrimination (below 100 Hz) and the noise in these measurements. Following this line of action, we will be able to quickly quantify the deposition of such inorganic salts with a resolution of, at least, below 10 μg.

## 4. Conclusions

The magnetoelastic resonance is an adequate phenomenon to be used in order to monitor, in real-time, the precipitation reaction of physiological inorganic salts, such as calcium oxalate ($CaC_{2}O_{4}$). The mass deposition of this salt onto the surface of the strip of the resonator gives rise to a shift of the resonance frequency that, by using the corresponding calibration curve, allows one to determine the mass amount deposited onto the resonator. Complementary information of the precipitation process arises from the measurement of the signal given by the amplitude of the detected magnetoelastic resonance, which is able to determine the time window in which the salt precipitation process occurs.

The corrosion resistant alloy used in this experiment ($Fe_{73}Cr_{5}Si_{10}B_{12}$) has been demonstrated to be as capable as the commercial Metglas^®^ 2826 alloy in monitoring precipitation reactions, with the advantage that no pre-treatment is required to prevent oxidation when used as a precipitation reaction sensor. In addition, the aspect ratio has been shown to play an important role in the sensitivity of the magnetoelastic sensor, leading to a more mass-sensitive sensor that allows the detection of smaller amounts of precipitate, and therefore a more accurate understanding of this kind of processes.

## Figures and Tables

**Table 1:** Magnetic and magnetoelastic parameters of the $Fe_{73}Cr_{5}Si_{10}B_{12}\;$ sample. The ones for Metglas^®^ 2826 alloy are also shown for comparison. Data taken from ref. [16].

Composition	$\mu_{0}M_{s}\;\left(T\right)$	$\lambda_{s}\left(ppm\right)$	∆E (%)	k	$E_{corr}$ (mV)	Corrosion Rate (μm/year)
$Fe_{73}Cr_{5}Si_{10}B_{12}$	1.12	14	17	0.41	47	0.035
$Fe_{40}Ni_{38}Mo_{4}B_{18}$ Metglas^®^ 2826 *	0.88	11	2.5	0.16	−427	23.4

^*^ Commercially available magnetoelastic ribbon [21,22].
